# Changing understanding of acromegaly epidemiology and early mortality risk

**DOI:** 10.1210/clinem/dgag150

**Published:** 2026-04-08

**Authors:** Daniela Esposito, Gudmundur Johannsson

**Affiliations:** Department of Internal Medicine and Clinical Nutrition, Institute of Medicine, Sahlgrenska Academy, University of Gothenburg, Gothenburg 40530, Sweden; Department of Endocrinology, Sahlgrenska University Hospital, Gothenburg 413 45, Sweden; Department of Internal Medicine and Clinical Nutrition, Institute of Medicine, Sahlgrenska Academy, University of Gothenburg, Gothenburg 40530, Sweden; Department of Endocrinology, Sahlgrenska University Hospital, Gothenburg 413 45, Sweden

**Keywords:** acromegaly, epidemiology, biochemical remission, mortality, morbidity

## Abstract

Epidemiologic research for rare diseases such as acromegaly is challenging due to low prevalence, heterogeneous data sources, and regional variability. Here, we review recent epidemiologic studies and provide a synthesis of the changing landscape of acromegaly and an overview of mortality rates and their determinants.

Over the past few decades, the reported incidence and prevalence of acromegaly have increased, likely due to improved diagnostic tools, earlier diagnosis, and more efficient management of the disease, leading to increased life expectancy. Available data suggest that the delay in diagnosis of acromegaly has progressively declined, and there is now a considerable increase in the rate of biochemical control—achieved in up to 90% of patients in some centers. This progress reflects improvements in disease management with the expanding use of multimodal and personalized treatment strategies. Consequently, mortality rates have substantially declined, approaching those of the general population. Despite these advances, most patients continue to be diagnosed only after acromegaly complications have developed. Comorbidities still have an independent and adverse effect on mortality and morbidity. Therefore, improved management of comorbidities is the optimal goal in the overall treatment of patients with acromegaly.

Acromegaly is a multisystem disease caused by excess secretion of growth hormone (GH) and its peripheral hormone, insulin-like growth factor 1 (IGF-I) (1, 2). Chronic GH and IGF-I excess lead to the typical clinical characteristics (1), which usually develop insidiously, and the disorder may remain unnoticed for several years. Systemic comorbidities are usually already present at the time of diagnosis, including cardiovascular, respiratory, metabolic, and musculoskeletal diseases (3, 4), resulting in impaired quality of life and excess mortality (1, 5).

The epidemiology of acromegaly has changed considerably over the past few decades. Early studies reported a prevalence ranging between 30 and 60 cases per million, whereas in more recent reports the prevalence has increased to 70 to 90 cases per million (6-10). Recent studies have also shown an increasing incidence of acromegaly (11, 12), which may be related to improved diagnostic techniques, including increased use of high-sensitivity GH and IGF-I assays and of brain imaging. This progress in diagnostic methods, together with improvements in disease management with increased survival rates, may have contributed to the rise in acromegaly incidence and prevalence (13). However, despite these advances, excess mortality remains a concern, especially in patients with suboptimal biochemical control and delayed diagnosis.

Epidemiologic research represents a challenge in rare diseases such as acromegaly due to limited sample sizes and regional variability in health care access. However, accurate epidemiologic data are necessary to improve our understanding of disease patterns and outcomes that can guide optimal resource allocation and to optimize diagnostic pathways as well as long-term management strategies. This review synthesizes current knowledge on the changing epidemiology of acromegaly and provides an overview of mortality rates and determinants of mortality.

## Epidemiology: a changing landscape

### Incidence and prevalence

Incidence and prevalence of acromegaly vary greatly among studies, as shown in Table 1 (7-10, 12-55). A systematic review and meta-analysis, including 32 studies worldwide from 1955 to 2016, showed that the pooled acromegaly prevalence was 5.9 (95% CI, 4.4-7.9) per 100 000 persons, while the incidence rate was 0.38 (95% CI, 0.32-0.44) cases per 100 000 person-years (56). However, considerable heterogeneity was found (with *I*^2^ of 99.3% for prevalence and 86.0% for incidence rate) that could be ascribed to differences in study design, geographical area, and different methods used for acromegaly identification. Notably, most studies (53.2%) were conducted in Europe, with the remaining studies in North America (19%), South America (9%), and Asia (19%) (56).

**Table 1:** Studies with acromegaly incidence and prevalence estimates

Author, year of publication	Country	Study period	Incidence (cases/1 000 000/year)	Prevalence (cases/1 000 000)	Number of patients
Roh et al, 2025 (14)	South Korea	2009-2019	46	—	459
Fauchier et al, 2024 (15)	France	2012-2021	7.6	104	7943
Robèrt et al, 2024 (16)	Sweden	1991-2018	5.1	—	1034
Rosendal et al, 2024 (17)	Denmark	1977-2021	4.6	108	889
Falch et al, 2023 (18)	Norway	1999-2019	4.7	83	262
Aagaard et al, 2022 (13)	Denmark	1992-2021	4.6	122	72
Arnardóttir et al, 2022 (19)	Sweden	1991-2011	3.7	—	698
Zaina et al, 2022 (20)	Israel	2000-2020	—	155	77
Yun et al, 2021 (21)	South Korea	2013-2017	4.2	32	1093
AlMalki et al, 2020 (22)	Saudi Arabia	2017-2019	—	6	195
Matsubayashi and Kawakami 2020 (8)	Japan	2013-2017	4.9	92	28 936
Park et al, 2020 (23)	South Korea	2010-2013	3.6	—	718
Wu et al, 2020 (24)	Taiwan	1997-2013	2.8	43	1195
Caputo et al, 2019 (25)	Italy	2012-2016	5.3	83	369
Gatto et al, 2018 (10)	Italy	2000-2014	3.1	69	74
Maione et al, 2017 (26)	France	1977-2012	—	17*^a^*	999
Al-Dahmani et al, 2016 (27)	Canada	2000-2013	3.8*^b^*	69	65
Aljabri et al, 2016 (28)	Saudi Arabia	2008-2015	—	33	10
Burton et al, 2016 (9)	USA	2008-2012	11	78	2241
Dal et al, 2016 (29)	Denmark	1991-2010	3.8	85	405
Fainstein Day et al, 2016 (30)	Argentina	2003-2014	9.2	141	19
López Gavilanez et al, 2016 (31)	Ecuador	2000-2014	1.3	19	48
Portocarrero-Ortiz et al, 2016 (32)	Mexico	1990-2012	—	18	2057
Agustsson et al, 2015 (33)	Iceland	1955-2012	—	137	53
Hoskuldsdottir et al, 2015 (12)	Iceland	1955-2013	7.7*^c^*	133*^d^*	52
Placzek et al, 2015 (34)	USA	2008-2012	—	42	757
Dal et al, 2014 (35)	Denmark	1991-2009	4.5	—	110
Tjörnstrand et al, 2014 (36)	Sweden	2001-2011	3.5	—	53
Gruppetta et al, 2013 (37)	Malta	2000-2011	3.1	125	52
Howlett et al, 2013 (38)	United Kingdom	1943-2011	—	46*^a^*	2572
Kwon et al, 2013 (39)	South Korea	2003-2007	3.9	28	1350
Vallette et al, 2013 (40)	Canada	1980-2011	—	17*^a^*	649
Almalki et al, 2012 (41)	Canada	1980-2008	—	29	130
Arosio et al, 2012 (42)	Italy	1980-2002	—	60*^a^*	1512
Mercieca et al, 2012 (43)	Malta	1979-2008	4.0	114	47
Cannavò et al, 2010 (44)	Italy	2008	—	97	64
Fernandez et al, 2010 (45)	United Kingdom	2006	—	86	7
Raappana et al, 2010 (46)	Finland	1992-2007	3.4	—	54
Carlsen et al, 2008 (47)	Norway	1999-2004	3.6	—	83
Bex et al, 2007 (48)	Belgium/ Luxembourg	2000-2004	1.9	40	418
Kauppinen-Mäkelin et al, 2005 (49)	Finland	1980-1999	4	—	334
Mestrón et al, 2004 (50)	Spain	1997-2004	2.1	34	1219
Ko et al, 1999 (51)	Hong Kong	1984-1992	3.8	—	34
Etxabe et al, 1993 (7)	Spain	1970-1989	3.1	60	74
Ritchie et al, 1990 (52)	Northern Ireland	1959-1984	4.1*^e^*	63*^f^*	131
Bengtsson et al, 1988 (53)	Sweden	1955-1984	3.3	69	166
Alexander et al, 1980 (54)	United Kingdom	1960-1971	2.8	53	164

^*a*^Estimate retrieved from Kerbel et al, 2023 (55).

^*b*^Incidence = 3.8 cases/1 000 000/year in 2013; incidence = 3.0 cases/1 000 000/year in 2000-2013.

^*c*^Incidence = 7.7 cases/1 000 000/year in 2005-2013.

^*d*^Prevalence = 133 cases/1 000 000 in 2013.

^*e*^Incidence = 4.1 cases/1 000 000/year in 1970-1983.

^*f*^Prevalence = 63 cases/1 000 000 in 1984.

Adapted from Rosendal C, et al. The changing landscape of acromegaly—an epidemiological perspective. *Rev Endocr Metab Disord*. 2024;25(4):691-705, with permission from Springer Nature (17).

Literature searches conducted to update table with additional references from 2024 and 2025. Literature search conducted November 2025.

PubMed: (“Acromegaly”[Mesh] OR acromegaly[tiab]) AND (incidence[tiab] OR “Incidence”[Mesh] OR prevalence[tiab] OR “Prevalence”[Mesh] OR epidemiol*[tiab]) AND (“2024/01/01”[PDAT] : “3000”[PDAT])

EMBASE (searches combined, duplicates removed):

1. (‘acromegaly'/exp OR acromegaly) AND (‘epidemiology'/exp OR epidemiology) AND [2024-2025]/py AND prevalence

2. (‘acromegaly'/exp OR acromegaly) AND (‘epidemiology'/exp OR epidemiology) AND [2024-2025]/py AND incidence

Regional differences play an important role in epidemiology, and prevalence of acromegaly is usually higher in countries with well-established national health care registries and centralized endocrine services. Additionally, the risk of acromegaly could be higher in some areas with a higher frequency of aryl hydrocarbon receptor-interacting protein germline mutations, although the overall incidence in these areas might not differ from that of other areas due to the low penetrance of this mutation (56).

The highest acromegaly prevalence estimates have been reported in Iceland (12, 33), Denmark (13), and Malta (37), with a prevalence of 133, 122, and 136 cases per million, respectively. These cohorts emanate from relatively small populations that have centralized care for rare diseases, likely reflected in high coverage of the disease in the population, and therefore, make them ideal for studying the incidence and prevalence of acromegaly.

The incidence and prevalence of acromegaly have increased over time. A recent single-center study from Denmark assessed changes in the epidemiology of acromegaly over time. Specifically, the mean prevalence increased from 69 to 96 to 116 cases per million, over three decades from 1992 to 2021 (13). On the other hand, the incidence remained stable at 4.6 cases per million. That study also reported changes in the clinical presentation, with a shift toward a milder phenotype. Data from Iceland also showed an increase in incidence from 1.2 cases per million per year (from 1955 to 1964) to 7.7 cases per million per year (from 2005 to 2013), which was associated with an increasing age at diagnosis (12).

Several factors may contribute to the apparent rise in acromegaly incidence and prevalence, such as advances in biochemical assays measuring GH and IGF-I, the increasing use of magnetic resonance imaging, and perhaps increased awareness of the disease. Moreover, improved treatment strategies, increased treatment options, and comorbidity management have improved survival rates, which have inflated prevalence due to prolonged life expectancy.

### Age at diagnosis and sex distribution

Acromegaly is usually diagnosed in the fifth decade of life (11). Epidemiologic studies have reported an increasing age at symptom onset and diagnosis over time (1, 13, 57). It is rarely diagnosed in children and young adults. In these cases, it usually manifests as pituitary gigantism, which usually is more aggressive, with higher GH and IGF-I levels, larger adenoma size at diagnosis, and a higher symptom burden (58).

Signs and symptoms of acromegaly are usually underrecognized, with diagnosis delayed by several years. Large national multicenter studies have reported a diagnostic delay ranging from 5 to 14 years (26, 42, 59). In a nationwide population-based study from Sweden specifically designed to study diagnostic delay, a mean delay of 5.5 years was found in a cohort of 603 patients with acromegaly between 2001 and 2013. However, 24% of patients were diagnosed with a delay of over 10 years (4). Available data suggest that diagnostic delay has progressively declined over time, whereas the age at diagnosis has progressively increased. This pattern has been observed in many recent studies and could be ascribed to a later symptom onset and a milder phenotype (59-61). Patients with milder disease forms were probably not diagnosed in the past, and the increased availability and use of IGF-I measurement may help to explain the observed increased incidence and prevalence.

There is a slight predominance of women diagnosed with acromegaly (52-60%) (62), with a recent meta-analysis showing a weighted percentage of female individuals with acromegaly of 53.3% (95% CI, 51.5-55.2%) (60). However, a shift from female predominance to a more even sex distribution has been reported (60). Women are usually older than men at diagnosis, with a median age difference of 3.1 years (95% CI, 1.9-4.4) (60). In addition, women are diagnosed with a 2- to 4.6-year longer diagnostic delay than men, despite consulting more physicians before diagnosis (62, 63). Women consistently present with more complications at the time of diagnosis (4). The likely explanation for these sex-related differences is that headache, amenorrhea, and sweating may be misinterpreted as menopausal symptoms in women (63). Another possible explanation is that women present with lower IGF-I concentrations at diagnosis, probably due to estrogen suppression of hepatic IGF-I production, resulting in less pronounced acromegaly features (64, 65). Implicit physician bias also may contribute to this sex disparity as observed in other diseases (66-68).

## Mortality: time trends

In the 1970s, Wright et al first reported increased mortality in acromegaly in comparison with the general population (69). The study included a cohort of 194 patients with acromegaly, with the mortality rates calculated in comparison with the general population of England and Wales, resulting in a standardized mortality ratio (SMR) of 1.9. Larger studies have later confirmed these results, showing a 2- to 3-fold increased mortality rate in acromegaly compared with age- and sex-matched controls (5, 70). The leading causes of death included cardiovascular disease, respiratory diseases, and, in some studies, malignancies (70-72). Over the past 2 to 3 decades, mortality in acromegaly has markedly declined (5, 73). This is likely related to improvements in surgical techniques for the removal of pituitary adenomas, the introduction of new treatment options, increased awareness, and better management of comorbidities such as hypertension, diabetes mellitus (DM), and dyslipidemia—all leading to improved disease control. Biochemical control is now achieved in most patients due to the use of multimodal and personalized treatment. In addition, the decreasing prevalence of hypopituitarism in patients with acromegaly has also contributed to a better prognosis (26, 73).

Recent registry studies have reported that SMRs in patients with well-controlled acromegaly may now be close to that of the general population (26, 29, 42). In a meta-analysis, including 16 studies published between 1970 and 2005, Dekkers et al showed that mortality was lower in studies published from 1995 onward (SMR, 1.62) in comparison with studies published before 1995 (SMR, 2.11) (70). In agreement with these data, a more recent meta-analysis of 26 studies with a total of 10 770 patients with acromegaly showed that the excess mortality has decreased over time from almost 2-fold in studies published before 2008 (SMR, 1.76; 95% CI, 1.52-2.04) to near normal in studies published after 2008 (SMR, 1.35; 95% CI, 0.99-1.85) (5). Cardiovascular disease was the main cause of death, accounting for nearly 50% of all deaths in the older cohorts. However, in recent epidemiologic studies, the main cause of death seems to have shifted from cardiovascular disease to malignancy. Ritvonen et al (74) analyzed causes of death in a nationwide cohort of patients from Finland with 20 years of follow-up. They showed that cardiovascular deaths decreased from 44% in the first decade to 23% in the second decade of follow-up, whereas cancer deaths increased from 28% to 35%, respectively. In agreement with these findings, Maione et al (26) studied mortality in the French Acromegaly Registry (*N* = 999) and showed that cancer was the leading cause of death, supplanting cardiovascular disease (34% vs 22%) (26). Similar findings were observed in an Italian survey of patients with acromegaly, in which 36% of patients died from malignancies and 28% from cardiovascular diseases (42), as well as in the Mexican cohort, in which 27% of patients died from malignancies and 9% from cardiovascular diseases (75). This shift in cause of death may be related to aging and follow similar trends also seen in the background population (5). Data on excess cancer risk in acromegaly remains controversial, with some studies showing an increased cancer incidence, especially due to colorectal cancer (76-79), yet not confirmed by other studies (80, 81). However, most recent studies have shown that cancer-related mortality in acromegaly is not increased in comparison with the general population (71). In the largest population-based study (82), including 1296 patients with acromegaly from Sweden, we showed that overall cancer risk was marginally increased but mortality due to malignancies was not different from the general population (SMR, 1.1; 95% CI, 0.9-1.4) (82). A possible explanation is that increased awareness of cancer in acromegaly may lead to more frequent screening for cancer, which is therefore diagnosed at an early stage. Additionally, the increasing proportion of patients with acromegaly achieving biochemical control may favorably impact cancer mortality (83).

In conclusion, mortality in acromegaly has declined over the last 2 to 3 decades, with life expectancy in well-controlled patients now approaching that of the general population. The leading cause of death has shifted from cardiovascular disease to malignancies. Advances in surgery, effective medical therapies, and better management of comorbidities such as hypertension, DM, and dyslipidemia have all contributed to reduced mortality. However, delayed diagnosis and persistent comorbidities can still negatively affect outcomes, underscoring the need for personalized, multidisciplinary management of acromegaly and its associated complications to improve long-term outcomes.

## Determinants of mortality

### Biochemical control and treatment strategies

Biochemical control is a strong determinant of mortality. There is convincing evidence that mortality in patients who achieve biochemical control is close to that of the general population (5, 84). Effective biochemical control also mitigates progression of major acromegaly-related cardiovascular and metabolic contributors to premature death (85). A systematic review and meta-analysis (5) of 26 studies and a total of 10 770 patients with acromegaly recently showed that mortality in patients with uncontrolled disease is increased 2-fold (SMR, 2.1; 95% CI, 1.5-2.9). Conversely, in patients with controlled disease, mortality is not significantly different from that of the general population (SMR, 0.9; 95% CI, 0.6-1.3) (5).

**Figure 1:**
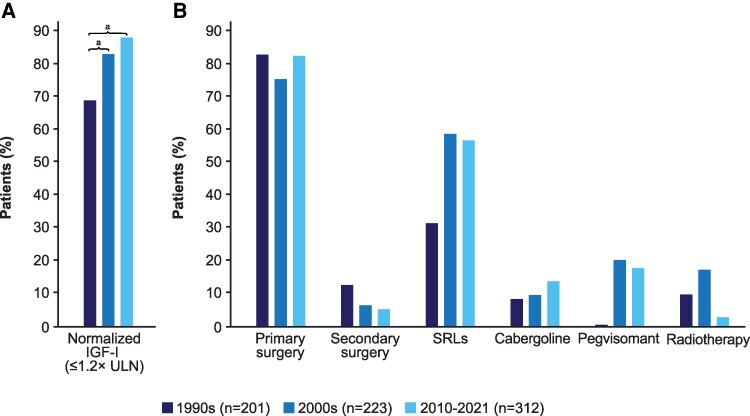
Evolution of (A) rate of biochemical control and (B) treatment strategies of acromegaly over 3 decades from 1990 to 2021. ^a^Statistically significant difference between study periods, binary regression with diagnosis decade 1990–1999 as reference. Abbreviations: IGF-I, insulin-like growth factor 1; ULN, upper limit of normal. Adapted from Rosendal C, et al. Changes in acromegaly comorbidities, treatment, and outcome over three decades: a nationwide cohort study. *Front Endocrinol (Lausanne)*. 2024;15:1380436, © 2024 Rosendal, Arlien-Søborg, Nielsen, Andersen, Feltoft, Klose, Andreassen, Bruun, Jørgensen, and Dal, under a Creative Commons Attribution License (CC BY) (89).

The proportion of patients achieving biochemical control differs across studies and time periods, reflecting changes in guideline criteria and resulting in a large variability of reported control rate, ranging from 40% to 90% (86, 87). Biochemical control in acromegaly is defined as normalization of age-adjusted IGF-I (≤1.0× ULN) (88). However, thresholds up to ∼1.2× ULN are sometimes pragmatically accepted in selected patients, although there is no strong evidence that such mild elevations are risk-free. Data from recent studies show that biochemical control has significantly increased over time, which may explain the improvement observed in survival rates (Fig. 1A) (89). In the French Acromegaly Registry, Maione et al (26) analyzed biochemical control by 4-year follow-up periods and found that the proportion of patients achieving biochemical control (including those with inactive disease and those with controlled disease with ongoing medical therapy) progressively increased from 43% before 2001 to 77% after 2010. A Danish nationwide study of 739 patients between 1990 and 2021 showed that the proportion of patients with normalized IGF-I increased over time, from 69% of patients diagnosed in the 1990s to 83% of those diagnosed in the 2000s and 88% of those diagnosed between 2010 and 2021 (17).

The improvement in biochemical control rates is likely related to better treatment over time. While pituitary surgery remains the cornerstone of management and first-line treatment, there has been a clear shift toward greater use of medical treatment, including somatostatin receptor ligands, dopamine agonists, and GH receptor antagonists. As a result, the use of radiotherapy has progressively declined due to its delayed efficacy and risk of long-term complications such as hypopituitarism (Fig. 1B) (89). Consequently, radiotherapy is currently reserved for selected patients with persistent disease after failure of surgery and medical therapy, with a trend toward personalized multimodal and pharmacologically driven treatment of acromegaly (88, 90). This approach is supported by early studies, which have shown a possible association between radiotherapy and excess mortality in patients with acromegaly (71). In a study based on the West Midlands Pituitary database, which contained details of 419 patients (241 female) with acromegaly, the use of radiotherapy was linked to an elevated SMR of 1.58 (95% CI, 1.22-2.04; *P* < .005) (91). Similarly, data from the Spanish Acromegaly Registry also supported this observation, showing that patients who died were more than twice as likely to have received radiotherapy than survivors (hazard ratio, 2.29; 95% CI, 1.03-5.08) (50). However, whether this excess mortality is due to the direct detrimental effect of radiotherapy itself or to a more severe, invasive, or treatment-resistant disease remains difficult to determine.

### Disease duration and diagnostic delay

Older age and prolonged exposure to GH excess increase the likelihood of irreversible complications. The diagnosis of acromegaly is usually made with a delay, ranging from 5 to 14 years across national registries (86). In the 1960s, Gordon et al (92) described a diagnostic delay of 10 to 20 years in a series of 100 patients with acromegaly. In the 1980s, Nabarro et al (93) reported a mean diagnostic delay of 9 years. More recent registry studies have described a diagnostic delay of approximately 5 to 6 years (4, 42, 94).

**Figure 2:**
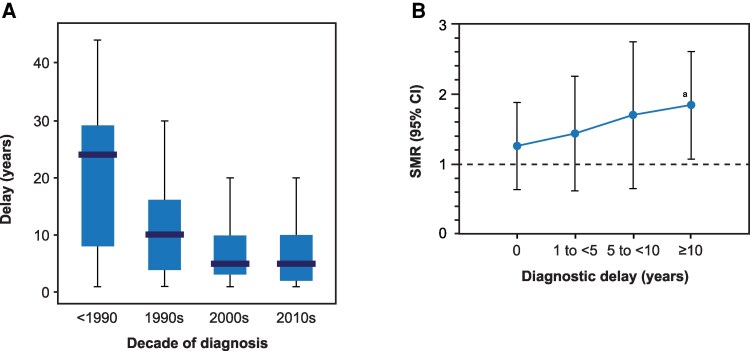
Diagnostic delay in acromegaly over time and its impact on mortality. (A) Estimated diagnostic delay in acromegaly displayed by the decade of diagnosis (*N* = 3000). Reprinted from Petrossians P, et al. Acromegaly at diagnosis in 3173 patients from the Liège Acromegaly Survey (LAS) database. *Endocr Relat Cancer*. 2017;24(10):505-518, © The authors, under Creative Commons Attribution 3.0 Unported License, https://creativecommons.org/licenses/ (57). (B) Diagnostic delay has an important impact on mortality. In a nationwide Swedish study, a significant increase in mortality was found in patients with a diagnostic delay of ≥10 years (SMR, 1.76; 95% CI, 1.12-2.65; *N* = 603). Mortality was not different from the general population in patients with a diagnostic delay of <10 years. ^a^*P* < .016. Abbreviation: SMR, standardized mortality ratio. Reprinted from Esposito D, et al. Prolonged diagnostic delay in acromegaly is associated with increased morbidity and mortality. *Eur J Endocrinol*. 2020;182(6):523-531, by permission of European Society of Endocrinology (4).

One of the largest multicenter studies based on a European acromegaly registry, including more than 3000 patients, showed that diagnostic delay progressively declined over time, from a delay of at least 20 years before 1990 to 5 years in the 2000s (Fig. 2A) (57). This improvement may be related to more awareness among clinicians and advancements in diagnostics, as well as the increased use of magnetic resonance imaging and incidental discovery of cases. However, efforts are still required to reduce diagnostic delays, as the majority of patients continue to receive the diagnosis only after comorbidities of the disease have developed. Notably, patients typically consult several specialists before a diagnosis of acromegaly is made—3 for men and 4 for women (63).

Diagnostic delay is associated with a poorer prognosis; thus, early diagnosis is crucial to improving long-term outcomes. In a nationwide study of 603 patients, our group showed that prolonged diagnostic delay is associated with increased morbidity and excess mortality. Specifically, patients receiving the diagnosis with a longer delay had more comorbidities at diagnosis and during the entire follow-up. Additionally, excess mortality was only found in the group receiving the diagnosis with a delay of at least 10 years (Fig. 2B) (4). The delay to diagnosis also seems to affect treatment strategies, as the use of radiotherapy is usually more common in patients with a longer delay in diagnosis, whereas the use of pituitary surgery is lower, which is probably due to an older age, higher number of comorbidities, and thus, higher surgical risks (4, 63).

### Hypopituitarism

**Figure 3:**
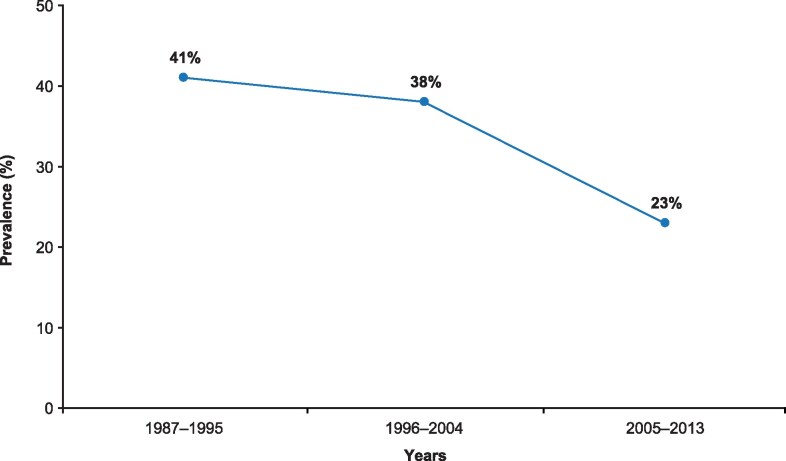
Change in prevalence of hypopituitarism in acromegaly over time (*n* = 1089) (73).

Hypopituitarism, resulting from the adenoma mass effect or as a consequence of surgical treatment or radiotherapy, affects approximately one-third of patients (26, 73). However, the rate of hypopituitarism has decreased over time. This is probably related to the improvement in surgical techniques and reduced use of radiotherapy and incidental and earlier diagnosis of milder disease. Our group studied time trends in the frequency of hypopituitarism in an unselected nationwide cohort of patients with acromegaly (*N* = 1089) and showed that the rate decreased from 41% in the first study period (1987-1995) to 23% during the last study period (2005-2013) (Fig. 3) (73). In line with these data, a single center study that included 409 patients with acromegaly between 1980 and 2019, showed a higher rate of pituitary deficiency before 2006 than after 2006 (67% vs 42%) (87).

It is well known that hypopituitarism itself is associated with excess mortality. However, data on the impact of pituitary dysfunction on long-term outcome in acromegaly are limited. Sherlock et al (95) analyzed mortality in a cohort of 501 patients with acromegaly and found that the relative risk was increased in patients who had adrenocorticotropic hormone–deficiency (risk ratio, 1.7; 95% CI, 1.2-2.5; *P* = .004), but not in those affected by gonadotropin- or thyroid-stimulating hormone-deficiency. In the adrenocorticotropic hormone–deficient group, higher replacement doses of hydrocortisone (>25 mg/d) were also associated with increased mortality than lower doses. Gonadotropin deficiency and GH deficiency in cured acromegaly have also been linked to adverse effects on body composition, bone health, and cardiovascular risk, although their independent impact on mortality remains uncertain (96).

### Comorbidities: hypertension and diabetes mellitus

Comorbidities such as metabolic and cardiovascular diseases have an important impact on mortality risk. Hypertension is one of the most prevalent comorbidities in acromegaly and is found in between 30% and 60% of cases in registry-based reports (97). Both GH excess and hypertension are factors considered responsible for the development of left ventricular hypertrophy, cardiomyopathy, valvular disease, and heart failure in acromegaly.

A single-center study followed 208 patients with acromegaly for a mean period of 13 years, during which 72 patients died (84). Patients who died during the period of observation were more likely to be older at diagnosis and have hypertension, diabetes, and/or hypopituitarism. Based on an international post-marketing registry of acromegaly, including 2090 patients treated with pegvisomant for a median period of 8.6 years, 64% had hypertension (98). Subjects with hypertension were older, had a higher BMI, and had an increased prevalence of diabetes mellitus (DM) and cardiovascular disease than patients without hypertension. A total of 78 deaths were recorded in the entire cohort: 68 in the group with hypertension (5.1%) and 10 in the group without hypertension (1.3%), with a mortality rate of 13.2 per 1000 patient years and 4 per 1000 patient years, respectively. Hypopituitarism and cardiovascular disease at study entry independently predicted mortality in patients with acromegaly and associated hypertension.

DM is also a common complication, occurring in one-third of patients, with a prevalence that progressively increases with longer exposure to GH excess. Older age and higher IGF-I concentrations at diagnosis, family history of DM, and increased body mass index are important risk factors for DM in acromegaly (99-101). It has been reported that pituitary macroadenoma (adenoma diameter ≥10 mm) and hypertension are more common in patients with acromegaly and associated DM than in those without DM, and that women with acromegaly could be at higher risk than men for DM (62, 102). In a multicenter study of 1512 patients with acromegaly, DM was an independent predictor of mortality (42). In a nationwide study from Sweden, mortality and cardiovascular morbidity were studied in 254 patients with acromegaly and concomitant DM in comparison to 532 patients without DM. After adjustment for disease duration, age, and multiple other confounding factors, overall mortality was 60% higher in the group with concomitant DM than in those without DM. Moreover, the group with DM had 2-fold higher cardiovascular mortality and 50% higher risk of cardiovascular disease (103).

### Sex differences in acromegaly

Sex differences in acromegaly have been increasingly recognized. Although earlier studies reported a female predominance, more recent data from population-based studies indicate a more balanced sex distribution (60). Women are usually older, experience longer diagnostic delays, consult more physicians before diagnosis, and have a higher burden of complications at diagnosis of acromegaly compared with men (60, 62, 63). Recent data also suggest worse long-term outcomes in women compared with men. Specifically, women seem to present with a worse metabolic profile, including a higher prevalence of insulin resistance, DM, and hypertension (104). In addition, women with acromegaly have been shown to have a lower socioeconomic status than men, including a higher use of social security benefits, an increased risk of early retirement, and lower rates of partnership (105). Interestingly, these differences seem to begin several years before the diagnosis of acromegaly.

Emerging evidence also suggests that women with acromegaly may have a higher mortality risk compared with men. In a recent nationwide cohort study, including 1884 patients with acromegaly and 94 200 controls from Korea, mortality risk was increased in women but not in men (106). In agreement with these findings, a multicentre study (107) including 118 patients diagnosed with acromegaly at age 65 or older in Spanish tertiary centres, have shown an increased SMR in women but not in men.

In conclusion, these findings suggest that sex differences in acromegaly are clinically relevant, with women being older at diagnosis, experiencing longer diagnostic delay, a higher burden of complications, and worse long-term outcomes compared with men.

## Changing perspectives and clinical implications

There is compelling evidence that the incidence of acromegaly has increased, likely due to improved access to better quality IGF-I assays, increased use of high-quality brain imaging with increased incidence of discovered pituitary incidentalomas requiring endocrine evaluation. Increased awareness of the disease has likely contributed to the increasing reported incidence of acromegaly. Furthermore, the increased prevalence of the disease may be due to the increased incidence, as well as improved life expectancy.

Results of observational studies strongly support the need to attain biochemical remission to potentially achieve a near normal mortality rate and to prevent comorbidities. Strategies to obtain this include pituitary surgery in high-volume centers and multidisciplinary treatment decisions on further treatment for those not achieving remission after surgery. Comorbidities related to the pituitary adenoma and its treatment, such as hypopituitarism, especially secondary adrenal insufficiency, and comorbidities related to GH excess, including hypertension and DM, have been highlighted as independent risk factors for death. The clinical implication of these observations in registry-based, single-center studies and national epidemiologic studies is the need for a higher standard of treatment for hypopituitarism, careful monitoring and treatment of hypertension, and strategies to prevent and optimally treat DM in patients with acromegaly.

## Conclusion

The incidence and prevalence of acromegaly have increased and this is more likely due to better and earlier diagnosis of the disease than a true increase in the incidence of somatotrophinomas. The increased prevalence is likely related to higher life expectancy due to earlier detection of the disease and more efficient management. Because comorbidities still have an independent and adverse effect on mortality and morbidity, improved management of hypertension, DM, and adrenal insufficiency is the optimal goal in the overall treatment of patients with acromegaly.

## Data Availability

Data sharing is not applicable to this article as no data sets were generated or analyzed during the present study.

## Abbreviations

DM: diabetes mellitus
GH: growth hormone
IGF-I: insulin-like growth factor 1
SMR: standardized mortality ratio
ULN: upper limit of normal
